# Small GTPases in Cancer: Still Signaling the Way

**DOI:** 10.3390/cancers13071500

**Published:** 2021-03-25

**Authors:** Paulo Matos

**Affiliations:** 1 BioISI—Biosystems & Integrative Sciences Institute, Faculty of Sciences, University of Lisbon, 1749-016 Lisbon, Portugal; paulo.matos@insa.min-saude.pt; 2 Department of Human Genetics, National Health Institute ‘Dr. Ricardo Jorge’, 1649-016 Lisbon, Portugal

In recent decades, many advances in the early diagnosis and treatment of cancer have been witnessed. However, cancerous diseases are still the second leading cause of death worldwide [1]. Moreover, the incidence of cancer in the last three decades has nearly tripled and some estimates indicate that this may increase by five-fold by 2030 [1,2]. Even more worrying, several epidemiological studies have indicated that the incidence of certain types of cancer is increasing sharply among young adults [1,3].

Despite a tremendous worldwide research effort, cancer is a highly complex and heterogeneous disease and the precise molecular mechanisms associated with its pathogenesis are still largely unclear.

It is commonly accepted that the development of tumors requires an initiator event, usually exposure to DNA damaging agents that cause genetic alterations such as gene mutations or chromosomal abnormalities, leading to deregulated cell proliferation. Although the mere stochastic accumulation of further mutations may cause tumor progression, it is now well established that the interaction of tumor cells with their surrounding microenvironment has an important role in modulating the epigenetic events that, together with genetic alterations, determine the initiation and progression of cancer [4].

In addition, changes in the tumor microenvironment (TME), such as abnormal vasculature, different immune cell infiltrates, hypoxic conditions, and variations in the composition of the extracellular matrix (ECM), are known to promote the selection of diverse malignant subpopulations within a single tumor mass [5]. This heterogeneity constitutes a major obstacle for the successful treatment of cancer, given that the administration of therapy often exerts additional selective pressure towards subpopulations with acquired resistance mechanisms [6].

Stromal cells in the TME, including fibroblasts, immune cells, and lymphatic and vascular endothelial cells, dynamically and reciprocally transmit information to tumor cells, and this two-way communication is known to be critical in promoting cancer progression [7].

Various cytokines, chemokines, and growth factors are involved in cell–cell communication within the TME. Moreover, linked to the recruitment of cancer-associated stromal cells, the TME becomes a mechanically complex environment, due to changes in ECM stiffness and architecture. This ECM remodeling helps to reprogram the phenotype of cancer cells, priming them for invasion and metastasis [8].

Mediating these communications are a myriad of cellular receptors that convey the microenvironmental stimuli into intracellular signaling pathways, triggering the dysregulation of epigenetic regulators, which synergize with acquired genetic alterations to promote cancer progression [9].

Downstream, most of these receptors are small GTPases of the RAS superfamily. These are low molecular weight proteins that cycle between an inactive GDP-bound and active GTP-bound state, functioning as molecular switches that regulate cytoplasmic signaling networks that control a diversity of cellular processes often dysregulated in cancer cells. Mutationally activated RAS genes encoding KRAS, HRAS, and NRAS—the founding members of this superfamily—were discovered in the early 1980s in human tumors, and now comprise the most frequently mutated oncogene family in cancer [10]. Given their broad involvement in cancer promotion and progression, RAS proteins, their regulators and downstream effectors have become of utmost importance in the development of anti-cancer therapies. However, after many unsuccessful attempts to target the small GTPases directly, they have often been classified as “undruggable” [11].

This prompted the development of new strategies targeting downstream effectors in RAS regulated signaling pathways. This approach has been somewhat successful, with several inhibitors of RAS effector kinases being approved for cancer treatment [12]. However, this approach has also faced unforeseen difficulties, such as selectivity issues, complex feedback mechanisms, and the development of drug resistance with the selection of resistant subpopulations of cancer cells [12]. However, while representing pioneering research, the RAS proteins are not alone in this demand.

The RAS superfamily of small GTPases expanded through the last 5 decades to encompass over 150 members. Based on their sequence, structural similarity, and functions in the cell, these proteins can be split into five smaller, evolutionally conserved subfamilies—RAS, RHO, RAB, ARF, and RAN [13]. GTPases in these five subfamilies integrate and relay extracellular and intracellular signals into an extensive network of signaling pathways, affecting almost all cellular processes, from gene expression and proliferation to cytoskeleton reorganization, vesicular trafficking, and ion transport. Overexpression or overactivation of certain members of all the subfamilies have been implicated in cancer initiation, promotion and progression [14,15].

RHO GTPases play central roles in numerous cellular processes, including cell motility, cell polarity, and cell cycle progression, by regulating actin cytoskeletal dynamics and cell adhesion [13,15]. Prior to acquiring malignant properties, tumor cells typically bypass any cell cycle checkpoints placed to suppress growth, resulting in uncontrolled cell proliferation and formation of a primary tumor. Microtubule and actin cytoskeleton reorganization during cell division are controlled by RHO GTPases and their dysregulated activity contributes to checkpoint evasion in cancer cells [15]. To promote transformation, cancer development, invasion and metastasis, tumor cells frequently hijack RHO GTPase activity, which is required for coordinated cell migration under physiological conditions [14,15]. In fact, the upregulation of several RHO GTPases has been detected in metastasis and late-stage tumors of different types [15]. Moreover, there is significant data indicating that the dysregulation of RHO GTPase activity has a profound impact in the coordination of the DNA damage response (DDR), impacting the DNA repair mechanisms that determine cancer cell survival or death [16].

The RAB family consists of approximately 70 members that play a critical role in the regulation of vesicular trafficking between different membrane-bound organelles [17]. Numerous studies have demonstrated that RAB GTPases and RAB-associated factors are major players in the TME, regulating the transport, adhesion, anchoring, and fusion of vesicles and the intracellular positioning and activity of signaling pathways in both stromal and tumor cells [18].

Aberrant expression of RAB proteins has been reported in multiple cancers, and mutations and/or abnormal post-translational modifications of these proteins dysregulate the overall trafficking network, promoting tumor progression and metastasis [18]. Conversely, some members of the RAB subfamily have been described as having tumor suppressive activity, inhibiting angiogenesis and promoting programmed cell death in certain tumor types [17,18].

The ARF subfamily of GTPases also participates in a large range of cellular processes, including organization of the cytoskeleton, the sorting of vesicle cargo, the recruitment of vesicle coat proteins, and the alteration of lipid membrane composition through the recruitment of specific enzymes and adaptor proteins in response to signals from the TME [19]. Dysregulation of some ARF isoforms has been shown to promote cancer formation and progression by stimulating tumor cell proliferation, namely through the activation of RAS-controlled mitogen-activated protein kinases (MAPK) [20].

The coordination between RAS and RHO GTPase signaling is determinant for tumor cell proliferation and survival and for cancer promotion [21]. Moreover, in epithelial cells, the abnormal but still interdependent signaling by RAB, RHO, and ARF proteins determines the type of cell–cell adhesion, coordinates collective cell migration and promotes epithelial–mesenchymal transition (EMT) during metastasis [22].

Finally, RAN GTPases control nucleus–cytoplasm export and the import of various molecules. RAN proteins also participate in the regulation of mitotic spindle assembly, thus, modulating chromosome spatial organization during cell division [19]. Perhaps because of this, the overexpression of RAN has been recently associated with increased cancer aggressiveness and the promotion of tumor cell proliferation, progression, and metastasis [23].

## Conclusions and Perspectives

Despite the limited success from almost three decades of research to find drugs that directly target dysregulated small GTPase activity in cancer, substantial progress has been made in understanding the biology, function, and signaling-crosstalk between many members of the RAS superfamily. However, new interplays, signaling pathways, and regulatory networks involving these molecular switches are being discovered every day. Therefore, understanding how the abnormal behavior of these proteins and their regulators or effectors allows cancer cells to adapt to the therapeutic inhibition of specific signaling events, will help to focus future efforts, and perhaps enable approaches that target small GTPase signaling networks at multiple levels.
